# Metal nanoparticles as a potential technique for the diagnosis and treatment of gastrointestinal cancer: a comprehensive review

**DOI:** 10.1186/s12935-023-03115-1

**Published:** 2023-11-19

**Authors:** Mohammad Roshani, Arya Rezaian-Isfahni, Mohammad Hassan Lotfalizadeh, Negar Khassafi, Mohammad Hassan Jafari Najaf Abadi, Majid Nejati

**Affiliations:** 1 Internal Medicine and Gastroenterology, Colorectal Research Center, Iran University of Medical Sciences, Tehran, Iran; 2 Student Research Committee, School of Medicine, Iran University of Medical Sciences, Tehran, Iran; 3 North Khorasan University of Medical Sciences, Bojnurd, Iran; 4 Anatomical Sciences Research Center, Institute for Basic Sciences, Kashan University of Medical Sciences, Kashan, Iran; 5 Research Center for Health Technology Assessment and Medical Informatics, School of Public Health, Shahid Sadoughi University of Medical Sciences, Yazd, Iran

**Keywords:** Metal nanoparticles, Gastrointestinal cancers, Therapy

## Abstract

Gastrointestinal (GI) cancer is a major health problem worldwide, and current diagnostic and therapeutic approaches are often inadequate. Various metallic nanoparticles (MNPs) have been widely studied for several biomedical applications, including cancer. They may potentially overcome the challenges associated with conventional chemotherapy and significantly impact the overall survival of GI cancer patients. Functionalized MNPs with targeted ligands provide more efficient localization of tumor energy deposition, better solubility and stability, and specific targeting properties. In addition to enhanced therapeutic efficacy, MNPs are also a diagnostic tool for molecular imaging of malignant lesions, enabling non-invasive imaging or detection of tumor-specific or tumor-associated antigens. MNP-based therapeutic systems enable simultaneous stability and solubility of encapsulated drugs and regulate the delivery of therapeutic agents directly to tumor cells, which improves therapeutic efficacy and minimizes drug toxicity and leakage into normal cells. However, metal nanoparticles have been shown to have a cytotoxic effect on cells in vitro. This can be a concern when using metal nanoparticles for cancer treatment, as they may also kill healthy cells in addition to cancer cells. In this review, we provide an overview of the current state of the field, including preparation methods of MNPs, clinical applications, and advances in their use in targeted GI cancer therapy, as well as the advantages and limitations of using metal nanoparticles for the diagnosis and treatment of gastrointestinal cancer such as potential toxicity. We also discuss potential future directions and areas for further research, including the development of novel MNP-based approaches and the optimization of existing approaches.

## Introduction

Gastrointestinal (GI) malignancies are the second leading cause of cancer death worldwide. GI cancers account for 26% of global cancer rates and 35% of all cancer-related mortality [1–13]. These malignancies involve various organs such as the anus, bile duct, gallbladder, stomach, colon, liver, and esophagus [14]. It is worth mentioning that some of the risk factors for the development of GI carcinogenesis include various genetic variants in oncogenes, mismatch repair genes, and tumor suppressors [12]. The buildup of cells caused by an imbalance between programmed cell death and cellular proliferation is a crucial cause of the development of GI cancers [15]. Multiple factors such as genetics, lifestyle (tobacco and alcohol consumption), and *Helicobacter pylori* infection play a role in GI carcinogenesis [9]. Early health concerns due to GI cancer inflict a staggering economic burden on the healthcare system and cancer patients [16]. The prognosis of gastrointestinal disease varies from person to person, based on the course of the disease at the time of diagnosis. It should be noted that if GI cancer is diagnosed early, patient outcomes can improve as they have the highest chance of being successfully treated. Therapeutic strategies include radiation, surgical procedures, and chemotherapy using various chemotherapeutic agents such as cisplatin, mitomycin, and docetaxel injection [9]. Most GI cancer chemotherapeutics are administered either orally or intravenously to achieve systemic distribution for effective treatment. However, due to lack of selectivity these drugs cause significant damage to rapidly proliferating normal cells. The major goal of targeted therapies is to target the chemotherapeutics to cancer cell which ultimately reduce the side effects. Nanoparticles are targeted either passively or actively to specific sites in GI cancer. A major problem in GI cancer chemotherapy is multidrug resistance which can be effectively circumvented via Mesoporous Silica Nanoparticles, Solid lipid nanoparticles, Polymeric nanoparticles and Magnetic nanoparticles. Poor aqueous solubility and low bioavailability of cancer chemotherapeutic can be effectively overcome by nanocrystals, albumin-based nanoparticles, liposomal formulation, Polymeric micelles, cyclodextrin and chitosan-based nanoparticles [17, 18].

Nanotechnology has drawn great scientific interest in current decades because of its appreciable functional and physical potentials [19–28]. The application of nanoparticles (NPs) in cancer therapy is feasible and affordable due to their physico-chemical characteristics, such as fine size, reduced toxicity, chemical composition, and large surface-to-volume ratio [29–34]. NPs have emerged as a breakthrough in treating numerous medical conditions, such as cancer. The utilization of NPs opens up innovative opportunities for detection (i.e. nanoimaging), nano-based drug delivery platforms (i.e. nanocarriers), and pharmaceutical applications [35–41]. Metal nanoparticles (MNPs) have gained specific attention among all the NPs because these have the potential to serve as multipurpose agents. MNPs mostly categorized based on the types of the metals. Gold, silver, iron and/or iron oxide, zinc, titanium, cerium oxide, nickel, copper, magnesium, barium, calcium, and bismuth-based metal NPs have been reported as a cancer treatment. The MNPs can be divided into noble and non-noble metals-based NPs. The noble MNPs such as gold and silver NPs is the metal from any of the several metallic chemical elements that have outstanding oxidation resistance, even at high temperatures. Non-noble MNPs such as magnetic and zinc oxide NPs despite their prone nature to oxidation have many advantages like they are low cost, abundant, and possess good conductivity [42]. MNPs are based on metals, such as gold, silver, copper, zinc, and used for various biomedical applications, from cancer diagnosis and enhancement of thermal ablation therapy to radiation therapy [43–45]. The use of various types of MNPs for the diagnosis and treatment of numerous cancers, such as lung cancer, breast cancer, and ovarian cancer has been reported [46–49]. NP-supported platforms provide better therapy with lower toxicity than conventional treatments. This is due to the robust electromagnetic (EM) field on the surface of MNPs, the extraordinary features arising from the optical characteristics, ease of production, surface chemistry, and surface functionalization [50, 51]. They provide good platforms for the target-specific and controlled delivery of chemotherapeutics in cancer therapy and allow simultaneous detection and management tailored to real-time chemotherapy control. Such NPs are applied alone or together with agents like tumor-specific antibody, peptides, DNA/RNA, or other small molecules to provide simultaneous targeting, imaging, and therapy, as a significant objective in cancer research and development [50]. Surface modification of metal oxide NPs has attracted extensive interest to minimize excess surface energy and thermodynamically stabilize NPs. This approach using green polymers such as polyethylene glycol can be utilized to mitigate NP agglomeration and increase NP stability [50, 52] .MNPs (e.g., Au/Ag/Pd) have highly adjustable optical characteristics that can be readily modified to desired wavelengths, making them the most likely developing trend in bioengineering materials applied probably as modern diagnostic tools and devices to treat critical illnesses [53, 54]. In addition, noble MNPs have aroused a big interest as potent NP for the thermal ablation of cancer cells by efficiently converting electromagnetic waves into heat [48, 55]. In this review, we have discussed the different preparation methods of MNPs. We have also provided a summary of MNPs application in treating different cancers with a focus on gastrointestinal cancer to better understand MNPs for desired therapeutic outcomes. One of the limitations of MNPs application is the biocompatibility and potential toxicity issues of metal nanoparticles. Metal nanoparticles have been shown to have a cytotoxic effect on cells in vitro. This can be a concern when using metal nanoparticles for cancer treatment, as they may also kill healthy cells in addition to cancer cells.

## Pathophysiology of gastrointestinal cancer

Gastrointestinal cancer refers to a group of cancers that affect the digestive tract, including the esophagus, stomach, small intestine, colon, rectum, and anus. These cancers are often referred to as colorectal cancers when they involve the colon or rectum. Gastrointestinal cancer is a major health problem worldwide, with high mortality rates and significant morbidity. Gastrointestinal cancers account for more than a quarter of global cancer incidence and a third of all cancer-related deaths [56].

The patho-physiology of gastrointestinal cancer is complex and not fully understood. It is thought to involve a combination of genetic, environmental, and lifestyle factors [57]. Genetic factors include inherited mutations in genes that regulate cell growth and division, such as the TP53 and APC (adenomatous polyposis coli) genes. In addition to genes, epigenetic modifications, include DNA methylation, microRNAs (miRNAs) and histone modifications play critical roles in GI tumorigenesis. Environmental factors include exposure to certain chemicals, such as tobacco smoke and industrial pollutants, as well as dietary factors, such as a high intake of red and processed meats. Lifestyle factors include a sedentary lifestyle and obesity. Several canonical oncogenic pathways deregulated in GI cancer, such as p53, PI3K/Akt, wnt/β-catenin, and nuclear factor (NF)-κB pathways [57].

The development of gastrointestinal cancer is thought to involve a series of stages, starting with the development of precancerous lesions, such as polyps, which can progress to cancer over time. As the cancer progresses, it can cause a variety of symptoms, depending on the location and extent of the cancer. Common symptoms, include diarrhea, wind, incontinence, abdominal pain, weight loss, changes in bowel habits, and blood in the stool. GI cancer is often treated with a combination of surgery, chemotherapy, and radiation therapy, depending on the stage and location of the cancer [58].

## Type of metal nanoparticles

### Silver nanoparticles

Silver nanoparticles (AgNPs) are highly effective against bacteria, viruses, and other eukaryotic microorganisms due to their excellent antimicrobial activity [59–63]. They are widely used as antimicrobial agents, in the textile industry, for water treatment, sunscreens, etc. [60, 64]. Allied to anti-bacterial, anti-fungal, and anti-viral, recent studies have shown anti-inflammatory, anti-angiogenic biological activities, and anti-cancer features of AgNPs. According to the multifunctional cytotoxic biological activities of biosynthesized AgNPs, they can be used as anticancer agents [65].

### Gold nanoparticles

Gold nanoparticles (AuNPs) have fascinated scientists for more than a century because of their versatile physical and chemical properties and are now widely used as theranostics, especially in cancer treatment and diagnosis. They are also used as laboratory tracers in DNA fingerprinting to detect the presence of DNA in a sample. They can detect aminoglycoside drugs such as streptomycin, gentamycin, and neomycin as potent bactericidal antibiotics. Gold NPs are used to identify cancer stem cells, making them a valuable tool for developing targeted cancer stem cell treatment [66, 67]. According to literature, gold nanoparticles have a strong contrast effect on computed tomography (CT) images, enabling the early detection of cancer. Gold NPs also possess a photothermal effect, which could be exploited to deliver photothermal therapy to cancer cells [68].

### Alloy nanoparticles

Metals can be synthesized into hybrid MNPs for synergistic effects in addition to their exceptional individual capabilities. Alloy NPs have more stable structures and better properties than monoMNPs, making them superior for biomedical imaging. Alloy nanoparticles are structurally different from their bulk counterparts. The improved properties of bimetallic alloy NPs have attracted scientific and industry interest. They show improved activity compared to conventional NPs. Bimetallic alloy NPs are cost-effective and stable alternatives with high activity and selectivity. However, due to safety concerns, alloy-NP-supported platforms have not yet been translated into clinical practice. The ability of MNPs in the ROS formation, oxidative stress induction, cytoskeletal stability disruption, and DNA degradation is a critical obstacle to their approval. Therefore, future studies should strive to improve the biocompatibility of such systems and gather additional information on safety issues and long-term effects [69].

### Magnetic nanoparticles

 Magnetic nanoparticles such as Fe3O4 (magnetite) and Fe2O3 (maghemite) have recently been introduced as unique materials due to features such as their safety and interaction with externally supplied magnetic fields. Nanomaterials with magnetic profiles are generally applied in various fields, including biomarker and cell separation, gene therapy, targeted cancer therapy, stem cell sorting, and manipulation, DNA analysis, and noninvasive imaging of human internal organs. Magnetic drug delivery is another powerful use of magnetic NPs in biomedicine that could be used to enhance conventional methods in cancer treatment [70].

### Selenium nanoparticles

Elemental selenium (Se) is an essential and rare micronutrient, while recently, Se nanoparticles (SeNPs) have attracted the attention of many researchers for their use as therapeutic agents, due to their higher biocompatibility and bioactivity, and lower toxicity than organic/inorganic Se compounds. Because of their connection with diverse moieties such as selenoproteins, selenomethionine, selenocysteine, and others, SeNPs play an important role in a variety of biological applications. They have anticancer, antidiabetic, antifungal, and antimicrobial activity, and are promising and excellent therapeutic agents in the fight against fatal diseases such as cancer, diabetes, and neurodegenerative diseases. In cancer cells, SeNPs activate selenoproteins and cause cell death, while protecting healthy cells by preventing ROS stress, chromosomal variations, and DNA damage. Chemosensitizing and chemoprotective properties are also present [71].

### Palladium nanoparticles

Palladium (Pd) is a highly valuable metal with exceptional catalytic, electroanalytical, and mechanical characteristics. Noble palladium nanoparticles (PdNPs) interact positively with biomolecules both within and outside of cells. In addition to the typical distinctive characterizations of metals, PdNPs have exceptional physicochemical features, including high thermal stability, outstanding photocatalytic activity, strong chemical stability, optical properties, electrical properties, and low cost. Their antimicrobial, antitumoral and antifungal of PdNPs have been proven. They could be used to develop novel photothermal agents, photoacoustic agents, gene/drug carriers, antimicrobial/antitumor agents, prodrug activators, and biosensors. Although the development of nanotechnology based on PdNPs for therapeutic applications is relatively new, their unique properties and significantly lower cytotoxicity mean that they are emerging as key players in the field of nanomedicine [72].

### Titanium nanoparticles

Titanium dioxide (TiO2) is an inorganic compound that has recently gained scientific attention due to its photoactivity. TiO2NPs are well-known for their attractive properties, including magnetic, optical, light weight chemical stability, low thermal conductivity, brightness, electrical conductivity, rheological, durability, high mechanical resistance, and biological properties. Additionally, these NPs possess biosafety, biocompatibility, and non-allergic reactions with the human tissues. Furthermore, TiO2NPs are easily available and cost effective. Regarding their unique properties, TiO2NPs are suitable and safe enough to be used in the field of medicine and dental applications. The combination of TiO2 nanoparticles with different molecules, antibodies or polymers has been studied for antimicrobial and anticancer photodynamic therapy [73].

### Zinc nanoparticles

Zinc nanoparticles have been found to have applications in biomedicine, agriculture, and other industries. Nano-ZnO is the most widely used type of ZnNPs due to its desirable properties, availability, low cost, stability, and neutral pH. It has been found to have antimicrobial, anticancer, antioxidant, antidiabetic, and anti-inflammatory activities, aid targeted drug delivery, and be used for diagnostic purposes. It has also been found to help mitigate zinc deficiency and stress tolerance in plants. In one study, Sharma et al. investigated the effects of ZnONPs on human liver cancer HepG2 cells and its possible pharmacological mechanism. They found that ZnONPs-exposed HepG2 cells had higher cytotoxicity and genotoxicity, which were linked to cell apoptosis mediated by the ROS triggered mitochondrial pathway. Mechanistic studies showed that the loss of mitochondrial membrane potential-mediated HepG2 cell apoptosis was mostly due to the diminution in mitochondrial membrane potential and Bcl-2/Bax ratios, as well as the activation of caspase-9. These findings provide insight into the mechanism of ZnONPs-induced apoptosis in human liver cells [74].

### Copper nanoparticles

**Fig. 1:**
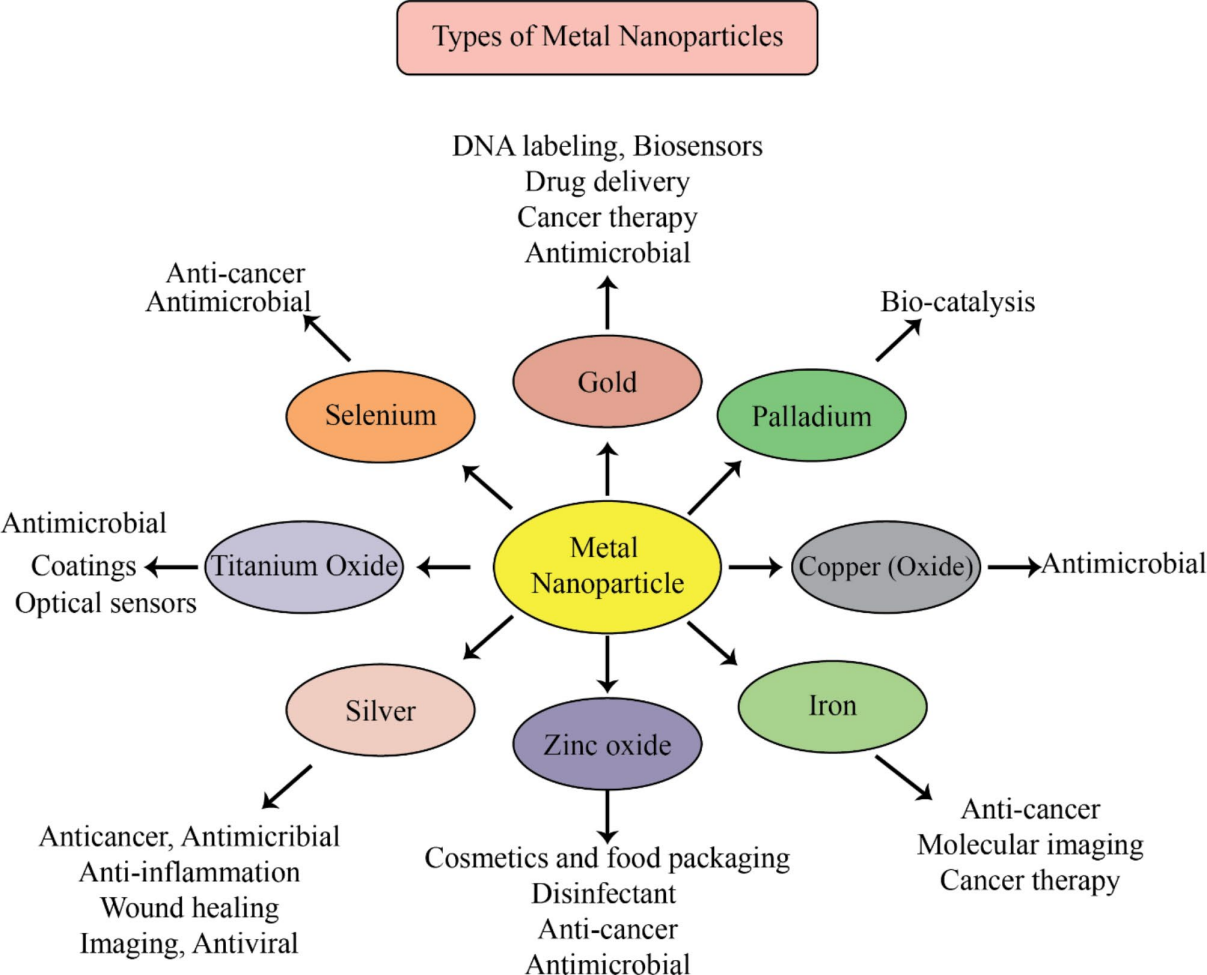
Types of metallic nanoparticles and their probable applications. This figure adapted from [77]

Copper (Cu) is an essential component of plant and animal metabolism, and is a soft, malleable, and easily bendable metal with high thermal and electrical conductivities. Compared to other transition metals such as platinum, silver, and gold, copper nanoparticles (NPs) are more cost-effective. In recent years, green synthesis of CuNPs has been emphasized, with broccoli green extract being described as a green and environmentally friendly precursor for one-pot biosynthesis. This method has been found to be beneficial in prostate cancer therapy [75]. Additionally, the cytotoxicity of a chitin-based silver and copper nanocomposite against human breast cancer (MCF-7) cells was investigated, with an inhibitory concentration (IC50) of 31 mg being found. Further findings revealed an increase in ROS production, decreased antioxidant enzyme activity, and membrane integrity degradation, confirming the cellular cytotoxic effect of the Cu–AgNPs-based nanocomposite [76]. Figure 1 illustrates some types of metal nanoparticles.

## Preparation methods for nanoparticles

**Fig. 2:**
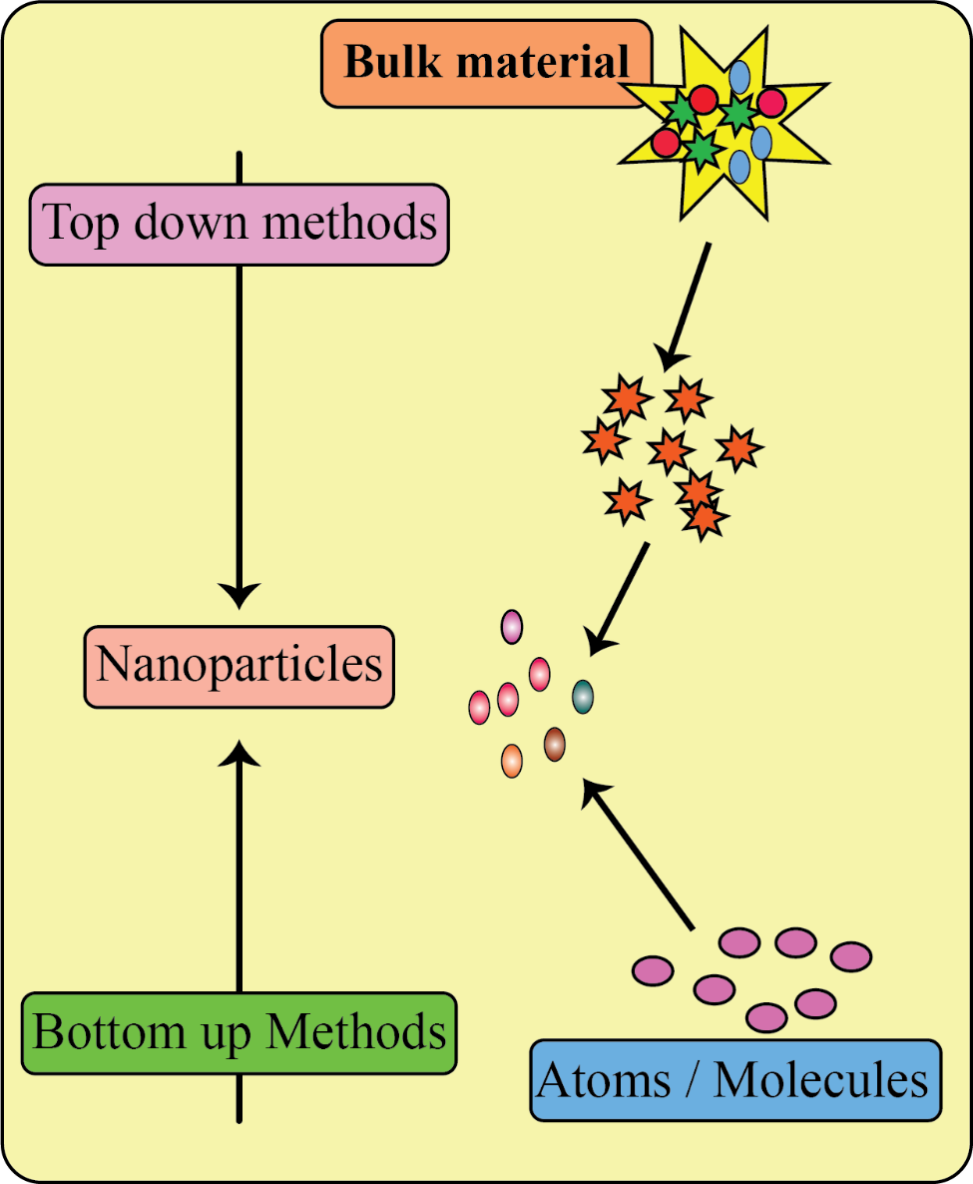
An overview of top-down and bottom-up approaches in the synthesis of NPs. This figure adapted from [90]

Metallic nanoparticles are produced using a variety of processes, which are classified into two categories: bottom-up and top-down approaches, see Table 1 [78–86]. Most of these approaches remain in the early stages of development, and challenges include NP stability and agglomeration, control of the crystallization process, and size distribution. In addition, the extraction and purification of the produced NPs remain critical for subsequent applications [87]. The primary distinction between the bottom-up and top-down methods is the starting material for synthesizing NPs. Top-down methods use various physical, chemical, and mechanical processes to break down bulk material into nanoscale structures or NPs, whereas bottom-up methods use nanospheres based on atoms/ions/molecules as the starting material for synthesis of nanoparticles (Fig. 2) [78, 79, 88, 89].

## Anticancer mechanisms of metal nanoparticles

The potential mechanisms of action of metal nanoparticles in cancer cells are not fully understood and are an active area of research. However, several mechanisms have been proposed, including:

1. Photothermal effect: Many metal nanoparticles, including gold nanoparticles, silver nanoparticles, and zinc nanoparticles, have been shown to have a photothermal effect when exposed to laser light. This means that they can absorb laser light and convert it into heat, which can be used to kill cancer cells through a process known as thermal ablation.
2. Free radical generation: Some metal nanoparticles, including gold nanoparticles and silver nanoparticles, have been shown to generate free radicals when exposed to light. These free radicals can damage cancer cells, leading to cell death.
3. Targeted drug delivery: Metal nanoparticles such as gold nanoparticles, silver nanoparticles, copper nanoparticles can be used as delivery vehicles to target drugs specifically to cancer cells. This can help to improve the effectiveness of chemotherapy and reduce the side effects of treatment [42, 91].
4. Antimicrobial and anti-inflammatory effects: Some metal nanoparticles, including silver nanoparticles and zinc nanoparticles, have antimicrobial and anti-inflammatory properties, which may contribute to their effectiveness in treating cancer.
5. Gene silencing: Metal nanoparticles such as gold nanoparticles can be used to silence genes involved in tumor formation through antisense DNA and short interfering RNA. Gene silencing is a process of altering gene expression on an epigenetic level to suppress genes implicated in tumor formation. This approach has been used to inhibit KRAS gene expression in colorectal cancer cell lines while leaving healthy fibroblasts unharmed. As another gene silencing option, small interfering RNAs (siRNAs) can be delivered to cells by using a platelet cell membrane-coated metal-organic framework nanoparticle. This approach has potential to be used as a cancer therapy [92–94].
6. Radiation therapy (RT) enhancement: Metal nanoparticles can be used to enhance the precision of radiation therapy and increase the effectiveness of treatment by decreasing the radiation dose and protecting healthy tissues from potential toxicity and injury. Various metal NPs have been used in recent RT research; however, silver and gold NPs surpass other metal NPs for radio sensitization applications in cancer imaging and therapy due to their high atomic number and mass-energy coefficient. Simultaneous treatment of ionization and hyperthermia is also effective, and when combined with metal and RT for cancer, response rates increase by 16–26%. Silver and gold NPs are the most commonly used for radio sensitization applications in cancer imaging and therapy due to their high atomic number and mass-energy coefficient. Combining photothermal therapy, IR sensitization, and targeted cytotoxicity with triangular silver NPs has been shown to selectively treat MDA-MB-231 breast cancer cells without harming nonmalignant MCF-10 A breast cells. Gold NPs and the histone deacetylase inhibitor SAHA have also been tested in 2D and 3D cancer cell cultures, and have been shown to significantly boost the potency of irradiation [42, 91].

**Fig. 3:**
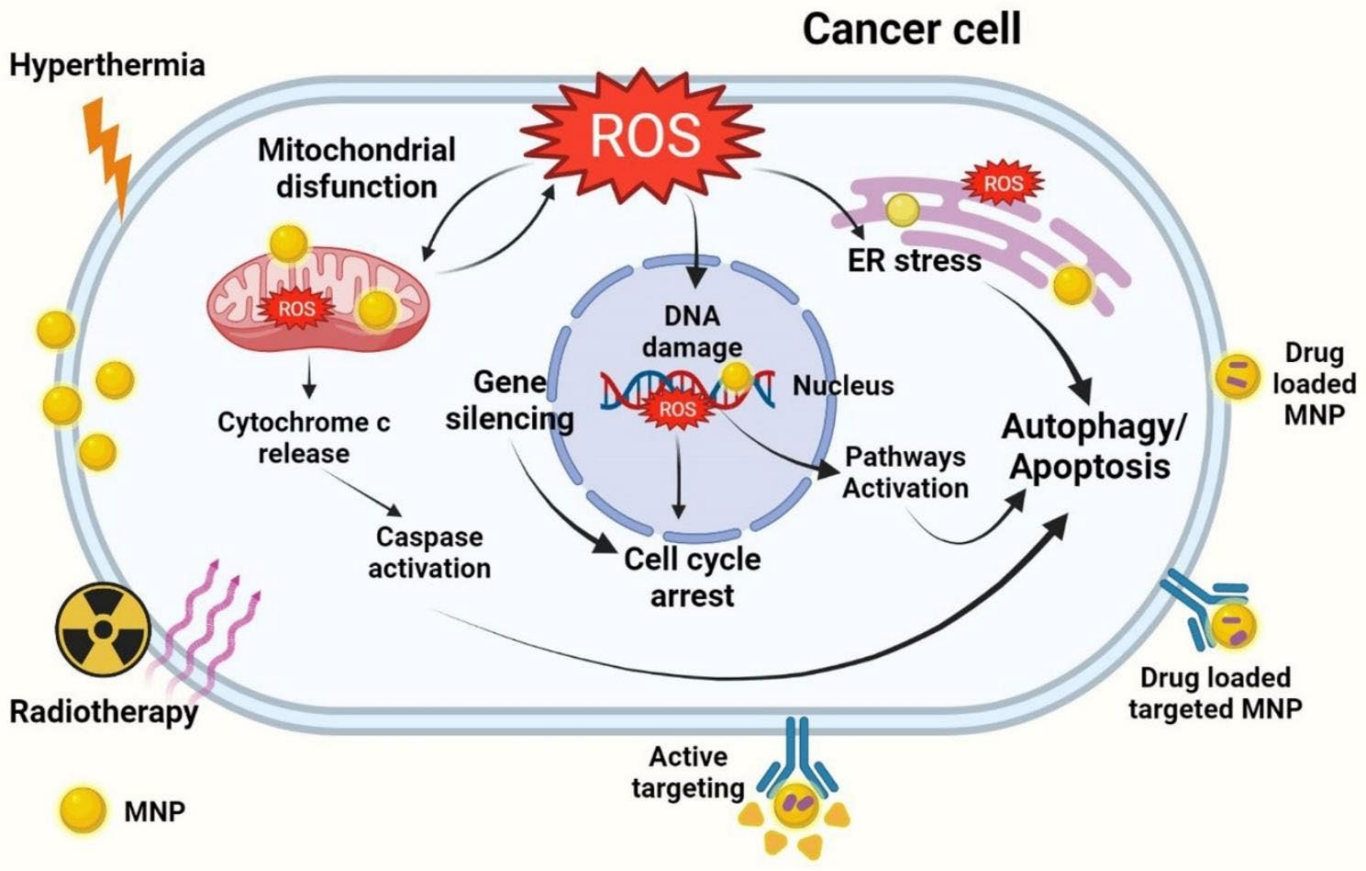
Anti-cancer mechanisms of action of MNPs. Metal nanoparticles possess anti-cancer activities *via* oxidative stress cause mitochondrial dysfunction, ER stress, autophagy and DNA damage, leading to apoptosis. arrest. In addition, metal NPs inhibit cell cycle to stop cancer proliferation. The figure was created with BioRender.com.

Overall, the potential mechanisms of action of metal nanoparticles in cancer cells are complex and depend on the specific type of nanoparticle and the cancer type. Further research is needed to fully understand the mechanisms by which metal nanoparticles exert their effects on cancer cells. The potential anti-cancer mechanisms of metal nanoparticles are shown in Fig. 3.

## Metal nanoparticles and gastrointestinal cancers

### MNPs suitable in gastrointestinal cancers

Gastrointestinal cancers are common solid malignant tumors, accounting for high mortality rates worldwide. MNPs have emerged as a promising tool for addressing this challenge, with a number of unique properties that make them well-suited for cancer diagnosis and treatment. Metal NP-based technologies provide more selectivity and sensitivity compared to the present instruments for early illness detection accessible of gastrointestinal cancer in the clinic, or they provide whole new capabilities that are not possible with conventional techniques. Although the selectivity and sensitivity of biomarker detection is improving, the laboratory diagnostic is not adaptable with clinical practice. The unique and tunable optical properties of metal NPs have revealed high potential in the expansion of biosensing and bioimaging techniques. Moreover, the anti-tumor effects of NPs for gastrointestinal cancers application can be further enhanced by surface functionalization or coating, and these NPs can be used for a variety of diagnostic, therapeutic, bioimaging, and prognostic purposes. MNPs that mostly have been studied for gastrointestinal cancer are includes gold NPs, silver NPs, zinc NPs. Therefore, more detailed information on the these specific types of metal nanoparticles have been provided as follow.

Gold nanoparticles: The non-reactive nature of gold makes it a noble element. Its resistance to chemical oxidation renders it impervious to degradation and corrosion. Thus, it can retain its form and luster for millennia. Gold NPs can be made in a variety of ways, including chemical, physical, biological, and green synthesis. The bottom-up and top-down approaches are used in all types. Gold NPs have a wide variety of biomedical applications due to their unique physicochemical characteristics. The anti-tumor effects of gold NPs can be further enhanced by surface functionalization or coating, and these NPs can be used for a variety of diagnostic, therapeutic, bioimaging, and prognostic purposes [42].

Silver nanoparticles: The primary mechanisms through which silver NPs function include ROS, oxidative stress, and DNA damage. ROS are essential for the survival of cells since they help to keep their internal balance in check. As a byproduct of cellular metabolism, ROS is a key player in signaling networks within cells. However, an excessive amount of intracellular ROS damages DNA, lipids, and proteins as a mechanism for silver NP-induced toxicity [95].

Zinc oxide nanoparticles: One of the most common metallic NPs in the world is zinc oxide. Zinc oxide NPs have received a lot of attention recently because of their ability to produce ROS when exposed to light. Particles of zinc oxide can be modified chemically to increase their photocatalytic efficiency as well as their ability to generate ROS by a variety of methods including doping with metals, polymer modification, and organic photosensitizing agents. The improved antibacterial and anticancer activity of modified zinc oxide NPs can be attributed to their increased ROS generation efficiency [96].

### Metal nanoparticles and gastric cancer

A gold nanoshell is a novel form of nanocarrier with a spherical core shape, a particle size of about 30 nm, and high efficiency in converting light to heat. The gold nanoshell can absorb near-infrared light at 785 nm to transform into thermal energy for hyperthermia of tumor *in vivo.* There are several advantages to this highly effective siRNA nanocarrier. The gold nanoshells can be better loaded with drugs because of their large surface area. Additionally, their unusual spatial arrangement can effectively prevent siRNA sequence degradation by enzymes. Gold nanoshells exhibit high biocompatibility and minimal cytotoxicity compared to other nanomaterials [97]. After irradiation with near-infrared, gold nanoparticles have a significant thermal effect. The high temperature destroys the thiol bond, releasing gene therapeutics. Simultaneously, the heat generated by the Au NPs can destroy tumor cells [98, 99].

Photothermal treatment has been shown to destroy primary tumors treated and trigger systemic antitumor immune responses. Additionally, the thermally-induced death of tumor cells can transfer tumor antigens to surrounding tissues, which can enhance the immune response when combined with immunotherapy [100]. Zhang et al. developed a multifaceted treatment strategy involving a gold nanoshell-based drug delivery system for the administration and selective photothermal release of genes targeting HER − 2 and unmethylated cytosine-phosphate-guanine oligodeoxynucleotide (CpG ODN) as an adjuvant, with the aim of treating gastric cancer. This study demonstrated the potential gene transduction and integrated therapeutic activity in vitro and in vivo by combining gene therapy, immunotherapy and hyperthermia, compared to monotherapy of each treatment. Gene therapy targeting HER − 2 showed a therapeutic effect at the molecular level. At the same time, photothermal treatment resulted in macroscopic ablation of tumor cells, and finally, immunotherapy could recognize and destroy cancer cells throughout the body [100]. Yun et al. synthesized AuNPs from an ethanloic extract of Vitex negundo (VN-AuNPs). They investigated their anticancer effects on AGS cells (a human gastric adenocarcinoma cell line) by examining cell viability, apoptotic morphological changes, apoptotic gene expression (caspase-3, Bcl-2, Bcl-XL, Bax), and reactive oxygen species formation (ROS). This synthesis of AuNPs from VN was environmentally benign, large-scaled up, and simple. This study observed increased ROS and MMP loss in the AGS cell line post VN-AuNP thrapy. In addition, treatment with VN-AuNPs increased the production of caspase-9, caspase-3, Bid, and Bax, while decreasing the production of Bcl-XL and Bcl-2, which may explain the increase in apoptotic cells in AGS cells exposed to VN-AuNPs [101]. Karuppaiya et al. synthesized biogenic silver nanoparticles (AgNPs) using an extract from the rhizome of *Dysosma pleiantha* and investigated their anti-cancer activity versus gastric cancer cell line (AGS) and breast cancer cell lines (MDA-MB-231, MDA-MB-453). The results showed that AgNPs-treated cells exhibited significant dose-dependent cytotoxicity. Compared to untreated breast cancer cells, there were alterations in shape, such as shrinkage and undifferentiated shape of the nucleus of MDA-MB-231 cells, and also elevated concentrations of AgNPs In addition, this study showed that the produced AgNPs caused DNA fragmentation and cells underwent apoptosis [102]. Therefore, the green AgNPs biosynthesized using the rhizome of *D. pleiantha* could be exploited in the production of new anticancer drugs [103]. Similarly, Mousavi et al. prepared AgNPs from the leaf extract of *Artemisia turcomanica* and investigated their effect on apoptosis in gastric cancer cell lines (AGS). According to biomass analysis and MTT assay, increasing amounts of commercial and phytosynthetic AgNPs boosted the inhibitory activity against the AGS cell growth. However, their method showed that phytosynthesized AgNPs inhibited cell proliferation at a lower dose than commercial NPs. Their findings showed that the biologically produced AgNPs promoted apoptosis and revealed a cytotoxic and anticancer activity against AGS cells in a dose-dependent and time-dependent manner. Moreover, this approach was environmentally friendly, safe, and cost-effective. As a result, green synthesized NPs can be an alternative to extracts in preventing or inhibiting cancer cell proliferation [104].

Selenium cations were concentrated to SeNPs using Kaempferia parviflora (black ginger) root extract and termed KP-SeNP. This nanoparticle was tested using various methods and found to be cytotoxic to AGS human gastric adenocarcinoma cells, but not to normal cells. It was determined that the anticancer effects of KP-SeNPs involve the upregulation of intrinsic apoptotic signaling markers and autophagy of AGS cells, specifically through the inhibition of PI3K/Akt/mTOR. Results from an AGS-cell xenograft model further validated these findings, showing that KP-SeNPs are biologically safe and can be used as a potential therapeutic agent for gastric cancer [105].

Zinc oxide nanoparticles (ZnONPs) have been found as a promising anti-cancer agent, but their role and mechanism of action in GC is yet unknown. Tang et al. synthesized Morus nigra-loaded zinc oxide nanoparticles (MN -ZnONPs). They tested their anticancer effects on AGS cells by measuring cell survival, apoptotic gene expression (Bax, Bcl-2, caspase-3, caspase-9), mitochondrial membrane potential (MMP) changes, and ROS production. The MN -ZnONPs synthesized in this study had a spherical shape, moderate size, and diverse functional groups. MN-ZnONPs elevated ROS production and lipid peroxidation, lowered MMP and antioxidants, and triggered cell cycle arrest in gastric cancer cells. A decrease in MMP is usually associated with an early stage of apoptosis. They conclude that MN -ZnONPs could be a therapeutic option for GC therapy. However, further research should clarify the potential significance of MN -ZnONPs for the clinical therapy of GC [106].

The effect of TiO2 NPs on apoptosis induction and invasion of gastric cancer cell line, MKN-45 was evaluated. Results showed that these NPs reduced cell proliferation and viability, and induced cell death. PEG-amorph TiO2 NPs increased cell invasion, while brookite BSA nanoparticles decreased cell invasion. These differences may be due to the effects of different configurations of TiO2 nanoparticles [107].

The studies employing MNPs for gastric cancer treatment are summarized in Table 1.

**Table 1:** The studies employing MNPs for gastric cancer treatment

Type of nanoparticles	Size	Major outcome	Targeting approach Passive or Active	Model ( In vivo, In vitro)	Type of cell line	Ref
Gold	30 nm	Well treatment effect on MFC tumor cell	Active	both	Mouse forestomach carcinoma cell	[100]
Zinc	200–700 nm	Increased apoptosis in AGS cell lines by enhancing pro-apoptotic/blocking anti-apoptotic proteins and arresting cell cycle	Passive	both	AGS gastric cancer cell	[106]
Gold	300-700 nm	Increased apoptosis	Passive	In vitro	AGS cells	[101]
Gold	50-80 nm	suppressed cell growth	Active	both	MKN7 and MKN74	[108]
Gold	100 nm	antitumor effects	Active	In vitro	AGS and L929	[109]
gold	151 nm	Detection of methylated RPRM DNA in patients with high sensitivity	Active	In vitro	KATO III	[110]
gold	128 nm	induces apoptosis	Active	In vitro	AGS, SNU-5, and SNU-16	[100]
gold	10–40 nm	enhanced cell apoptosis	Passive	In vitro	A549, HT29 and AGS	[111]
gold	50 nm	suppressed tumor growth	Active	both	NCI-N87	[112]
gold	20 to 30 nm	Cytotoxicity effect	Passive	In vitro	MKN45, AGS, and KATO III	[113]
gold	5–60 nm	induce apoptosis	Passive	In vitro	AGS	[114]
silver	76 nm	cytotoxic potential	Passive	In vitro	AGS	[103]
silver	22 nm	apoptosis induction o	Passive	In vitro	AGS	[104]
silver	5–50 nm	induce apoptosis	Passive	In vitro	AGS	[115]
silver	20–50 nm	scavenging of free radicals and iron chelating activity	Passive	In vitro	AGS	[116]
iron oxide	67.3 nm	Prevention of metastasis	Passive	both	SGC-7901	[117]
Lanthanide	670 nm		Passive	In vitro	MKN45 and HeLa	[118]
gold	10, 20 and 40 nm	decreased invasion activity	Passive	In vitro	SGC-7901	[119]
gold	45.97 ± 4.67 nm	destroyed cell spindle morphology, ruptured cell 562 membranes	Passive	both	MCG803	[120]

### Metal nanoparticles and colon cancer

Colorectal cancer is a crucial etiology for death and morbidity globally. Therefore, new techniques are continuously being researched in addition to existing therapies. Cancer therapy based on nanotechnology is a novel approach that is considered one of the most promising research opportunities for colorectal cancer. Various MNPs, like Au, Ag, copper (Cu), iron oxide, zinc oxide (ZnO), etc., can be used for the targeted anticancer drug delivery and diagnostic purposes in colorectal cancer. Among the noble metals, Au and Ag NPs are considered to be the most common NPs utilized in targeted anticancer drug delivery in colorectal cancer.

K. Chaturvedi et al. synthesized AuNPs and AgNPs with the aid of Pleurotus sajor-caju extraction and demonstrated cytotoxic effect versus HCT-116 (human colon cancer cell line). Pleurotus sajor-caju extract is an anticarcinogenic and immunostimulant agent and also contains high levels of microelements, like Cu, found on the synthesized NPs surface [121]. Compared to the control, the exposed HCT-116 cells lost morphology and cell adhesiveness and shrank, with apparent fragmentation of DNA. After HCT-116 cell exposure, Ag NPs were found to have the highest cytotoxicity, followed by PS extract and Au NPs. Ag NPs had the most significant effect on cellular ROS generation, but PS extract and Au NPs had nearly comparable impacts. Similar changes were observed in cancer cell lines such as HeLa and Hep-2 cells after treatment with Ag NPs prepared from plant extracts. This method ensures the imprinting of the active substance on the surface, making it more capable of targeting the colon cancer cell line, ushering in a new era of herbal therapy [121].

Arya et al. synthesized a surface-modified Au NPs containing inositol hexaphosphate (IP6), and *Jacalin* (a plant lectin) which could enhance the apoptotic activity of IP6 against HCT-15 cells (a human colon cancer cell line). IP6-loaded jacalin-pectin-gold nanoparticles (IJP-GNPs) were evaluated for their ability to destroy HCT15 cells in numerous assays. Dosage-dependently and time-dependently, IJP-GNPs caused strong apoptotic effects due to the cell cycle arrest at the G0/G1 phase and provoked ROS production in HCT15 cells. In vitro studies support the interaction response of IP6, PGNP, and jacalin that probably target and suppress tumorigenesis. All data indicate that IP6-loaded nanoparticles could manage the colon cancer [122]. In another study, cellular prion protein (PrPC) aptamers (Apt) anchored on AuNPs were synthesized to targeted delivery of doxorubicin (Dox) to colorectal cancer as PrPC-Apt-loaded Dox-oligomer-AuNPs (PrPC-Apt DOA). PrPC glycoprotein on the cell surface can be overexpressed in colorectal cancer. The results showed that compared with free Dox therapy, the oligomer PrPC-Apt DOA delivered Dox successfully to colon cancer cells and significantly enhanced apoptosis and decreased proliferation in CRC cells. This suggests the possibility of using PrPC-Apt DOA as CRC treatment [123].

However, there are conflicting data on the pharmacokinetics of AuNPs, such as biodistribution, toxicity, and clearance in the treatment of cancer, particularly colorectal cancer [46]. Leve et al. investigated the role of citrate-capped gold nanoparticles (GNP) on the structure and function of the apical junctional complex (AJC). They also assessed whether these NPs could enhance the therapeutic effect of cetuximab in different CRC cell lines. Based on the obtained results, GNPs regulate the barrier function of the apical tight junction (TJ) and increase paracellular permeability. The TJ is responsible for both barrier function and signaling pathways in cancer development. As intercellular junctions provide a barrier to monoclonal antibody delivery during treatment, GNPs are suitable for drug delivery. The results also showed that cetuximab-GNP promotes cell death of invasive CC cells. In addition, using lower doses of cetuximab than the therapeutic dose by this approach may reduce adverse side effects and treatment costs [124].

A study examines the use of CuNPs for the treatment of colon cancer. The authors found that copper nanoparticles had a strong cytotoxic effect on colon cancer cells in vitro and also had a potent inhibitory effect on the proliferation of colon cancer cells [125].

Recently, Klebowski et al. investigated the effect of PtAu and PdAu NPs in proton therapy (PT) on colon cancer cell lines (SW480, SW620, and HCT116) and normal colon epithelium cell line (FHC). Their results showed that the NPs-assisted PT had a better anticancer effect than PT alone, but there was no significant difference in the radiosensitizing properties between the nanocomplexes. The data also showed that the treatment was more selective for cancer cells, as normal cell viability was only slightly affected. Overall, the combined approach of proton irradiation and bimetallic nanocomplexes resulted in a significant inhibition of cancer cell proliferation and viability [126].

Javed et al. constructed AgNPs based on the aqueous extract of *M. arvensis* (mint) of the *Lamiaceae* family and investigated their growth inhibitory activity for HCT116 colon cancer cells. They also examined the effects of temperature, pH, and reactant concentration on the production of AgNPs biologically prepared from the aqueous extract of *M. arvensis*. The results of this study provided experimental evidence for understanding the synergistic influence of physicochemical reaction parameters on nanoparticle formation and their ability to inhibit cancer cell development. The apoptosis assay with Annexin V demonstrated that biologically prepared AgNPs triggered apoptosis in a dosage and time-dependent manner. Cell cycle studies on HCT116 cancer cells showed that AgNPs interrupted cell division in the sub-G1 phase, suggesting that AgNPs affect cell survival and inhibit future proliferation [127]. These results explain how physicochemical factors interact to enhance the phytosynthesis of biocompatible AgNPs and overcome the limitations of conventional chemotherapeutic therapies for colon cancer cells.

One study examined the anticancer properties of SeNPs synthesized by the probiotic strain *Lactobacillus casei* ATCC 393. It was found that the nanoparticles were less toxic than other forms of selenium and had cancer-specific antiproliferative activity and apoptosis-inducing potential. When administered orally, selenium nanoparticle-enriched *L. casei* was found to be more effective in attenuating the growth of colon carcinoma in mice than the isolated nanoparticles or *L. casei* alone, suggesting a potential additive effect [128].

Vigneshwaran et al. synthesized TiO2NPs using *Lactobacillus* and tested their cytotoxic potential in HT-29 cells, a human CRC cell line. The results suggest that TiO2 is capable of inducing cytotoxicity in those cells by generating intracellular ROS and activating the intrinsic apoptotic pathway [129]. The impact of industrially prepared TiO2NPs on HCT116 colon cancer cell line using an in vitro model was assessed. The size and surface charge of the particles were altered by high-energy ball milling, and the particles were found to be cytotoxic and genotoxic, inducing significant apoptosis and genotoxic cytotoxicity. *In silico* analysis showed that Sod1, Sod2, p53, and VLDR proteins had a significant role in determining the cytotoxicity. The particles also revealed noteworthy antibacterial activities [130].

**Table 2:** The studies employing MNPs for colorectal cancer treatment

Type of nanoparticles	Size	Major outcome	Targeting approach (Passive or Active)	Model ( In vivo, In vitro)	Type of cell line	Ref
gold	37 nm	Cytotoxic effect	Passive	In vitro	HCT-116	[121]
silver	23 nm	Cytotoxic effect	Passive	In vitro	HCT-116	[131]
gold	520 nm	drug delivery	Passive	In vitro	CT-116	[124]
gold	20–200 nm	The electroporation-GNPs method could create an opportunistic context for colon cancer therapy	Passive	In vitro	HT-29	[132]
gold	7.9 ± 1.7 nm	apoptotic activity	Passive	In vitro	HT-29 and Caco-2	[133]
Silver	31 ± 8 nm	apoptotic activity	Passive	In vitro	lines HT-29 and Caco-2	[133]
gold	20–30 nm	Anticarcinogenic effect	Passive	In vitro	HCT-116	[134]
gold	20–40-nm	antiproliferative and genotoxic effects	Passive	In vitro	HCT-15	[135]
gold	3–5 nm	Inhibition of colon cancer cell growth	Passive	In vitro	HT-29	[136]
gold	50 nm	increased apoptosis	Passive	In vitro	HT-29	[137]
gold	28 nm	effective treatment drug against a chronic inflammatory condition that progresses to malignancy	Passive	In vitro	HT29	[138]
gold	50 nm	induction of apoptosis	Passive	In vitro	HCT-116	[139]
gold		inhibit the tumor formation	Passive	In vitro	HCT-15	[140]
gold	36 nm	induced oxidative stress and apoptosis	Passive	In vitro	SW-480	[141]
gold		reducing the relative tumor volume	Passive	both	CT26	[142]
silver	< 100 nm	suppress the growth of cancer cells	Passive	In vitro	HCT116	[143]
silver	2–10 nm	Increase Cytotoxicity	Passive	In vitro	HCT116	[144]

### Metal nanoparticles and pancreatic cancer

Pancreatic cancer is rare compared to other cancers, yet it is among the deadliest solid cancers with a poor prognosis. This cancer is the third main etiology for death in the US and is expected to become the second leading cause of death by 2030. The development of therapeutic applications using nanotechnology may provide new options for treating pancreatic cancer. Many studies have reported promising outcomes from using nanoparticles in treating pancreatic cancer (Table 3). For example, Saha et al. found that AuNPs decrease the growth and metastasis of pancreatic stellate cells (PSCs) and pancreatic cancer cells (PCCs) by the elimination of bidirectional communication due to changes in cell secretome. They found that AuNPs decrease the production of major nodal proteins in both cell lines and shift active PSCs in a lipid-rich quiescent state. AuNPs also decrease the deposition of matrix, increase, and limit tumor development in the in vivo model of orthotopic co-implantation by inhibiting PSC activation. Therefore, AuNPs have the potential to be used as a mean to efficiently study bidirectional interactions in the microenvironment of tumor and suppress tumor development through their curative activity [145]. Huai et al. investigated whether AuNPs can sensitize PCCs to gemcitabine (a chemotherapeutic agent) and whether AuNPs administration can counteract gemcitabine-induced adverse effects in gemcitabine-resistant PCCs, such as ERK MAPK pathway activation, stem cell formation and EMT signaling. The AuNPs were found to sensitize tumorigenic gemcitabine-resistant pancreatic cancer cells to gemcitabine. AuNPs also inhibited MAPK signaling, attenuated EMT effects, and reduced gemcitabine-induced stem cell formation. AuNP treatment also blocked the signaling pathway for cancer spread. Therefore, the AuNPs make pancreatic cancer cells more sensitive to gemcitabine *via* defending them from gemcitabine-mediated adverse effects [146]. Recently, AuNPs have emerged as attractive vehicles for gene delivery and RNA interference technologies due to their varying sizes, surface properties, and various functional capabilities. They lead to effective gene knockdown in cells and tissues with minor cellular damage or off-target effects [147–149]. SiRNA is a potential therapeutic approach to suppress the overexpression of oncogenes and remedy mutations in tumor suppressor genes as a post-transcriptional gene regulation process for treating incurable diseases such as cancer. However, the in vivo utilization of siRNA is limited by its inefficient distribution to target systems [150]. A. Jensen et al. investigated the ability of covalently functionalized AuNPs with tightly packed, highly oriented small interfering RNA duplexes to inhibit the expression of Bcl2Like12 (Bcl2L12) oncoprotein, p53 inhibitor and effector caspase, which are highly expressed in GBM. In vivo, the functionalized Au NPs successfully crossed the blood-brain barrier (BBB) and blood-tumor barrier (BTB), neutralized Bcl2L12 expression, and glioma cells sensitized to therapy-mediated death by increasing effector caspase and p53 activity. Moreover, the functionalized AuNPs effectively reduced tumor burden and delayed progression of cancer in glioma-carrying mice without causing complications [148]. Similarly, Hao et al. evaluated the ability of miRNA-AuNPs to control cell processes by raising endogenous level of miRNA in the PC-3 (human prostate cancer cell line). Human miR-205 was used as a proof-of-concept miRNA due to its tumor-suppressive activity in the human prostate through the down-regulation of PRKCε (Protein kinase C epsilon type), and its expression is significantly reduced in PCa cell lines [151, 152]. The miR-205-AuNPs therapy reduced the PRKCε expression up to 52% when compared to cells exposed to controls loaded with non-targeted sequences. This approach also significantly inhibited cancer cell proliferation and migration. Overall, the potential of polyvalent nucleic acid-loaded NPs to introduce cells without the support of polymers and cationic lipids, combined with their proven activity as endogenous miRNAs, makes them a fantastic tool to evaluate miRNA performance and an exciting technology candidate for miRNA-based therapy [151]. Another study investigated whether gold nanocluster-assisted NGF-siRNA delivery (GNC-siRNA) provides effective NGF gene silencing and pancreatic cancer therapy. They found that the GNC-siRNA combination improved siRNA stability in serum, prolonged the lifespan of siRNA circulation in the blood, and improved cellular absorption of siRNA and tumor accumulation. This complex significantly reduced tumor formation in three pancreatic tumor models by knocking down NGF expression (subcutaneous, orthotopic, and patient-derived xenograft models) [153]. Their research has demonstrated a simple but highly successful method of inhibiting pancreatic tumors, providing a unique treatment approach for pancreatic cancer.

In recent decades, chemotherapy has been considered the first choice in the cancer therapy. However, because of severe complications of direct administration of chemotherapeutic agents, physicians prescribe the lowest dose of chemotherapeutic agents that may interfere with cancer treatment. AuNPs have recently received much attention due to their unique ability to deliver chemotherapeutic agents. Imanparast et al. used PEGylated hollow gold nanoparticles (HAuNP) as nanocarriers for mitoxantrone (MTX), a chemotherapeutic agent that is also a photosensitizer and radiosensitizer. They reported that PEGylated gold mitoxantrone NPs increased therapeutic efficacy in cancer. HAuNPs are an attractive choice for drug delivery due to their hollow structure, large internal and external surface area, high biosafety, biocompatibility, and functional capacity [154]. Qiu et al. developed a novel multifunctional theranostic nanoplatform using gold nanocages (AuNCs) modified with hyaluronic acid(HA) and coupled with an anti-GPC1 antibody, oridonin (ORI), gadolinium (Gd), and Cy7 dye, which they termed GPC1-directed NCs (GPC1-Gd-ORI@HAuNCs-Cy7 NPs). They tested its capabilities in MRI and NIRF imaging and its therapeutic activity in treating pancreatic cancer under both in vivo and in vitro circumstances. This nano-system was temporally stable and exhibited fluorescence/MRI properties. Moreover, when comparing to ORI and ORI-NPs, NCs targeting GPC1 dramatically decreased the viability and increased apoptosis of BXPC-3 and PANC-1 (pancreatic cancer cell lines overexpressing GPC1) but not 293 T cells (pancreatic cancer cell line negative for GPC1) [155]. Thus, GPC1-Gd-ORI@HAuNCs-Cy7 NPs represent a potential theranostic basis to co-detect and successfully treat pancreatic cancer [155].

Additionally, researchers such as Zielinska and co-workers assessed the ability of AgNPs to kill pancreatic cancer cells and identified the molecular mechanism behind this effect. Results showed that AgNPs decreased viability, proliferation and caused death of pancreatic cancer cells in a size- and concentration-dependent manner. Cellular uptake of AgNPs resulted in apoptosis, autophagy, necroptosis and mitotic catastrophe, which were associated with increased pro-apoptotic protein Bax and decreased level of anti-apoptotic protein Bcl-2, as well as increased levels of tumor suppressor p53 protein and necroptosis- and autophagy-related proteins [156].

**Table 3:** The studies employing MNPs for pancreatic cancer treatment

Type of nanoparticles	Size	Major outcome	Targeting approach (Passive or Active)	Model ( In vivo, In vitro)	Type of cell line	Ref
gold	20 nm	prevented migration and colony formation	Passive	In vitro	PANC-1, AsPC-1, MIA PaCa-2 and HPAF II	[146]
gold	430 nm	inhibits the tumour progression	Passive	In vitro	Panc-1	[153]
gold	4.5 nm	Uptake and excretion dependent on the cell line	Passive	In vitro	BxPC-3	[157]
gold	20 nm	inhibits the upregulation of stem cell markers	Passive	In vitro	PANC-1, AsPC-1, MIA PaCa-2, HPAFII	[158]
gold	630 nm		Passive	In vitro	Panc-1	[159]
gold	150 nm	Induce apoptosis	Passive	both	PANC-1, SW1990	[160]
gold	10–100 nm	Promoted the apoptosis	Active	both	PANC-1, BXPC-3, SW1990	[161]
gold	0.8 and 15 nm	cytotoxic effect	Passive	both	PANC-1	[162]
gold	10–100 nm	prevented viability and boosted apoptosis	Active	In vitro	PANC-1, BXPC-3, and SW1990	[155]
gold		inhibit desmoplasia and tumor growth	Active	both	PANC-1	[163]
gold	1–2 nm	increased efficacy of traditional chemotherapeutics.	Active	In vitro	PANC-1	[164]
gold	20 nm	Inhibits Tumor Growth	Passive	In vitro	PSCs	[145]
silver	2.6 and 18 nm	there was a greater cytotoxic effect on the PANC-1 cells than the non-cancerous cells	Passive	In vitro	PANC-1	[165]

### Gold nanoparticles (AuNPs) and hepatocellular carcinoma

Hepatocellular carcinoma (HCC) is in the second position of cancer incidence globally, with a five-year survival rate of 18.4%. HCC currently accounts for over 25% of cancer-related deaths worldwide [166]. In recent years, significant progress has been made in developing and using NPs to detect and manage HCC cancer (Table 4). However, there are several limitations that must be overcome. The miR-221/p27/DNMT1 pathway is a promising target in cancer therapy due to its frequent dysregulation in HCC and its critical role in regulating cell growth, migration, viability, and angiogenesis. Approximately 60% of hepatocellular carcinomas exhibit aberrant miR221 signaling, and there is an association between poor prognosis and miR221 signaling activity in patients receiving sorafenib as a chemotherapeutic agent [167–169]. Sorafenib (a kinase inhibitor) is an antineoplastic drug that has been shown to prolong overall survival in people suffering from advanced HCC [170]. However, in some patients, the predicted therapeutic efficacy is not achieved [171, 172]. In a study by Cai et al., the anti-tumor activity of sorafenib in combination with Au NPs loaded with anti-miR221 was determined versus HCC cells. Anti-miR221 is a specific miR221 inhibitor that stimulates p27expression while suppressing DNMT1 expression, potentially increasing the efficacy of sorafenib in HCC. Their results showed that treatment with AuNPs-loaded anti-miR221 and sorafenib alone had little influence on inhibiting the HCC cell proliferation. In contrast, the combination of sorafenib and AuNPs-loaded anti-miR221 synergistically inhibited the proliferation of HCC cell lines. Consequently, AuNPs-loaded anti-miR221 can act as a potential chemosensitizer to enhance the chemotherapeutic efficacy of sorafenib in HCC [173].

Ma et al. developed SM5-1-anchored AuNPs (Au-SM5-1 NPs) for their potential anticancer activity against HCC under both in vivo and in vitro conditions. The SM5-1 (humanized mouse monoclonal antibody) suppressed the proliferation of tumor cell and induced the apoptosis. SM5-1 selectively binds to P230, a membrane protein highly expressed in various cancer types, such as HCC, breast cancer, and melanoma [174, 175]. The Au-SM5-1 NPs were effective in inhibiting the tumor growth rate in HCC in a dosage-dependent and time-dependent manner compared with SM5-1 alone. The tumor suppression rate of Au-SM5-1 NPs increased by 39.64% ± 4.87% after 31 days in tumor-bearing mice. According to quantitative reverse transcription PCR (qRT-PCR), NOX1 and DUOX2 genes were downregulated, but the P21 gene was upregulated significantly. In anticancer drugs, these genes trigger cell apoptosis [174]. Similarly, Ebrahim et al. investigated the additive or synergistic potential of photothermal therapy (PTT) using laser-irradiated AuNPs with a low dose of sorafenib to reduce the adverse effects of sorafenib. The cytotoxic effect and apoptosis rate of HepG2 were examined after incubation with sorafenib and/or non-irradiated or laser-irradiated AuNPs. The results showed that AuNPs and sorafenib decreased the viability of HepG2 cells, and the cytotoxicity of AuNPs and sorafenib was associated with increased LDH release in the culture medium. When sorafenib was combined with laser-irradiated AuNPs, the most synergistic cytotoxic activity on HepG2 growth was observed, indicating the possibility of replacing high lethal doses of sorafenib with lower doses in combination with PTT [176]. Lu et al. demonstrated that the combination of AuNPs-encapsulated microbubbles (AuMBs) with sonoporation-assisted delivery could significantly improve the delivery efficiency of AuNPs in a human liver cancer line both in vitro and in vivo and also improve the megavoltage (MV) radiosensitization. Sonoporation-induced cavitation increases the intracellular AuNP level, causes DNA damage, and eventually apoptosis. Physical variables (size/shape) of AuNPs and cell line type influence AuNP-mediated radiosensitization. Irradiation conditions (dose/dose rate, radiation parameters) also have a major impact [177, 178]. In a mouse model of xenograft, the VEGFR2 (vascular endothelial growth factor receptor 2)-targeted AuMBs in combination with sonoporation could significantly delay progression of tumor. However, the precise biological processes and AuNPs-cell interactions have yet to be completely understood, the research offers initial evidence of MV radiosensitization using AuNP sonoporation in liver cancer cells [177].Young Park et al. studied the anti-metastatic properties of four *Maclura tricuspidata* (MT) extracts (root (MTR), leaf (MTL), stem (MTS), and fruit (MTF)) for cytotoxic effects on hepatocellular carcinoma cells. MT belongs to the family *Moraceae* that is widely used as a traditional herbal remedy due to its various benefits. It has been shown that AuNPs can reduce metastasis in HCC, thereby increasing the efficacy of therapy. In addition, MT-AuNPs have been found to inhibit cell migration, invasion, MMP-9 and PLD activity, and other proteins associated with EMT. Migration and invasion are critical for the spread of HCCs. Thus, the results provide compelling evidence that MT-AuNPs are beneficial in reducing HCC metastasis and may be a potential treatment option [49].

Choi et al. investigated the antitumor effect of AuNPs (40 and 80 nm) functionalized with cationic branched polyethylenimine (BPEI), anionic lipoic acid (LA), or neutral polyethylene glycol (PEG) against C3A cells (a human hepatocellular carcinoma cell line) with and without human plasma protein corona (PC). Cytochrome P450 (CYP) enzymes are crucial in the inhibition or bioactivation of various cytotoxic agents and host sensitivity to anticancers. All no PC AuNPs had an inhibitory effect on CYP 3A4 activity, regardless of surface charge and size but PC, on the other hand, recovered its action besides PEG-AuNP. Time-dependent cellular absorption of AuNP enhanced in addition to 40 nm BPEI-AuNP, whereas PC reduced it in addition to 80 nm PEG-AuNP. These findings can be helpful to better understand the interactions of AuNP with critical bio-processes and relevant molecular pathways in HCC, probably resulting in the creation of more effective curative targets for diagnosis and management [179].

**Table 4:** The studies employing MNPs for hepatocellular carcinoma treatment

Type of nanoparticles	Size	Major outcome	Targeting approach (Passive or Active)	Model ( In vivo, In vitro)	Type of cell line	Ref
gold	1–100 nm	delayed tumor growth	Passive	In vitro	Huh7	[180]
gold	35 nm	enhanced the tumor suppressors	Passive	In vitro	HepG2	[181]
gold	1–100 nm		Passive	In vitro	HepG2	[182]
gold	19 ± 0.37 nm	decreased tumor growth	Passive	In vitro	HepG2	[183]
gold	13 nm	inhibit proliferation of HCC cell	Passive	In vitro	HepG2 and Huh7	[173]
gold	13 nm	high tumor uptake	Passive	both	Hep3B and HepG2	[184]
gold	10 nm	suppress hepatocellular carcinoma growth	Passive	both	HepG2	[185]
gold	83.7 ~ 103.6 nm	Anti-Metastatic Effect	Passive	In vitro	HepG2 and SK-Hep-1	[186]
gold	40 and 80 nm	Inhibited Cytochrome P450 3A4 Activity and Molecular Mechanisms Underlying Its Cellular Toxicity	Passive	In vitro	C3A	[179]
silver	30.71 nm	Induced ROS and cell apoptosis in HepG2 cell line	Passive	In vitro	HepG2	[187]
silver		focal necrosis and inflammatory cell infiltration	Passive	In vivo	mice	[188]

### Metal nanoparticles and esophageal cancer

Light-absorbing MNPs with the maximal resonance at a determined wavelength can be unified with a near-infrared light source to create a photothermal activity in the particles. This activity converts electronic vibrations to heat at the particle surface [47]. Various formulations of near-infrared absorbing NPs have been created to produce particles safe for biological tissue, with the capability of carrying the heat to tumors while causing minimal damage to neighboring normal tissue [189]. Examples are nanocages, nanorods [190, 191], nanoshells [192–194] and gold/gold sulfide (GGS) NPs [195]. Localized heat has been shown to induce cellular damage, resulting in a therapeutic procedure that is extremely elegant, focused, and effective. When nanoparticles are heated to relatively high temperatures, a number of consequences occur, including denaturation of target cells, bubble formation, and mechanical stress on the target cells, all of which result in cell death [196, 197]. Li Y et al. investigated the effect of near-infrared light on photothermal ablation in human esophageal cells and in a rat model of esophageal duodenal anastomosis after treatment with chitosan-supported gold/gold sulfide NPs (CS-GGS). The results showed that after irradiation with near-infrared light, both cancer cells and malignant tissue absorbed more AuNPs and were largely ablated. They also found that irradiating healthy esophageal cells with near-infrared light with no NPs had no harmful consequence. Although further research is needed, CS-GGS nanoparticles may be an ideal and exciting option for the subjects suffering from esophageal cancer without the need for surgery [198].Copper oxide nanoparticles (CuO NPs) are frequently used in paints, plastics, and textiles, because of fascinating structural, biological, and pharmacological properties. Cu-based products have been licensed for human use in the USA since 2008 [199, 200]. These nanoparticles are actively researched as they have unique characteristics with various physiological and metabolic roles [201]. Several tumor cells have shown significant toxicity to these nanoparticles, including lung adenocarcinomas (A549), monocytic leukemia cells (THP-1), and human esophageal squamous cell carcinomas (KYSE30 cells) [202, 203]. Nakhaeepour et al. developed a simple, rapid, and environmentally friendly method for preparing CuO NPs with anticarcinogenic, antibacterial, and photoluminescent properties (PL) using live cells, cell lysate supernatant (CLS) and protein extracts of Vibrio luminescent species. They proved that the CLS technique was more successful in synthesizing CuO NPs fabricated using copper sulfate (CuO NPs-1) and copper nitrate (CuO NPs-2). The cytotoxicity of CuO NP -1 against the KYSE30 esophageal cancer cell line was dose-dependent, but it had no increased cytotoxicity against normal human dermal fibroblast (HDF) cells. CuO NPs can cause both DNA damage and cell death in malignant KYSE30 cells, demonstrating their anticancer properties. This may be due to the CLS containing bioactive chemicals that serve as encapsulants for the CuO NPs. Moreover, the antibacterial potential of CuO NP -1 versus determined pathogens has been demonstrated, suggesting that the present CuO NPs have multifunctional properties [204]. Martin et al. determined the potential toxicity of widely used AgNPs over the production of basement membrane (BM), integrin interactions, and tissue integrity using models of immortalized, nontransformed lung epithelial cells [205]. They observed gross morphologic changes induced by AgNPs during 3D growth and noted a substantial increase in glandular structures (acini) in response to AgNP exposure. This increase in the development of 3D acini was associated with features associated with the malignant transformation of mammary epithelial cells, suggesting that AgNP exposure may induce comparable unregulated growth features in other cell and tissue types [206, 207]. Their results showed that AgNPs affect epithelial cell BM synthesis and adhesion interaction. The expression of CD44 (a cell surface glycoprotein with a role in cell adhesion, cell-cell interactions and migration) was significantly higher in AgNP-treated cells compared to control cells. It remains to be determined whether these changes can cause epithelial disorganization that persists over time and contributes to the affected tissue to diseases such as fibrosis or cancer. Similarly, Scoville et al. found enhanced CD44 gene expression in vivo in lung tissue of mice treated for 24 h with 20 nm citrate stabilized AgNPs compared to controls [208]. These results support the theory that AgNP exposure selects cells that can withstand stress *via* CD44-mediated signaling pathways. In addition, they observed a correlation between the pathogenic potential of AgNP and the dysregulation of critical signaling nodes such as FAK (increased), caveolin-1 (decreased), Src, and ERK1/2 (increased) [205]. These studies shed light on the processes behind the physiological perturbations of the epithelium triggered by AgNP.

### Clinical trials using MNPs for GI cancer

MNPs are explored in early and clinical studies for detection, diagnosis and treatment of several diseases. MNPs have received widespread attention based on their unique material and size-dependent physicochemical property which are impossible with organic NPs. FDA-approved metal nanoparticle–based nanomedicines in clinical use have been shown to enhance the bioavailability and efficacy of drug delivery systems, while at the same time reducing side effects due to their properties like improved targeted delivery to active cellular uptake. By adjusting their sizes and shapes, surface chemistry and doping techniques, the designed MNPs can decompose rapidly under specific physiological conditions and are thereby easily absorbed by various metabolic pathways without affecting the healthy tissues. MNPs approved for cancer therapy by FDA or EMA and clinical trials such as Iron oxide NPs in Prostate cancer (NCT02033447), Magnetic iron oxide NPs in leukemia (NCT01411904), Gold nanoparticle with iron oxide silica cell in atherosclerosis (NCT01436123 ) [209].

Despite challenges that mentioned above, currently there are few registered clinical trials in clinicaltrials.gov website that are using metal nanoparticles for the treatment of gastrointestinal cancer patients.Recently Hoegen and el al. published an article related to a phase 2 clinical trial (NCT05027711) entitled “Magnetic Resonance-guided Adaptive Stereotactic Body Radiotherapy for Hepatic Metastases (MAESTRO)”. This MAESTRO trial aims to assess the potential advantages of adaptive, gated MR-guided SBRT (Stereotactic body radiotherapy) compared to conventional SBRT for treating hepatic metastases. SBRT is a local treatment method for patients with liver metastases, but it is limited by the proximity of radiosensitive organs at risk. MR-guided radiotherapy (MRgRT) is expected to improve SBRT by providing better soft-tissue contrast for target identification and the ability to adjust treatment in real-time. The primary endpoint is the non-inferiority of MRgRT regarding hepatobiliary and gastrointestinal toxicity, and secondary outcomes include local, locoregional, and distant tumor control, progression-free survival, overall survival, and quality of life. The trial also evaluated dosimetric parameters, morphological and functional changes in MRI, and potential prognostic biomarkers. The authors discussed that MRgRT is both highly cost- and labor-intensive and MAESTRO trial is a randomized study that seeks to determine the effectiveness of MR-guided, on-table adaptive and gated SBRT for dose escalation in hepatic metastases located near radiosensitive organs at risk. The results of the trial will provide higher-level evidence of the dosimetric and potential clinical benefits of this treatment [210]. A cohort study (NCT04682847) was lunched to develop and evaluate radiotherapy with Iron Oxide Nanoparticles (SPION) on MR-Linac for primary and metastatic hepatic cancers. A phase I/II trial, NCT04789486, aims to determine the safety and efficacy of gadolinium based nanoparticle, Activation and Guidance of Irradiation X (AGuIX), used in conjunction with MR-guided SBRT in the treatment of pancreatic cancer and lung tumors [211].

### Limitations and challenges in the clinical translation of MNPs for GI cancer

While metal nanoparticles show promise as a potential technique for the diagnosis and treatment of gastrointestinal cancer, there are several limitations and challenges that need to be considered.

Another limitation is the potential for metal nanoparticles to accumulate in the body over time. Metal nanoparticles have been shown to accumulate in organs, such as the liver and spleen, and can potentially have long-term health effects. This needs to be carefully monitored and minimized in order to ensure the safety of metal nanoparticle-based treatments. A further challenge is the need to optimize the size, shape, and surface chemistry of metal nanoparticles in order to maximize their effectiveness for cancer diagnosis and treatment. Different sizes, shapes, and surface chemistries of metal nanoparticles can have different effects on cancer cells and may be more or less effective for different types of cancer. Developing methods to optimize the size, shape, and surface chemistry of metal nanoparticles for cancer diagnosis and treatment is an active area of research. Other limitations in the clinical translation of MNPs in the treatment of GI cancer include incomplete biodegradation and elimination, a long drug development process, failure of effective drug loading inside NPs, challenges in drug internalization and release, challenges in cellular uptake, and the failure to translate the in vitro results to in vivo studies [212–214].

Additionally, the scale-up of nanoparticle synthesis, especially for metals, is however very challenging. Stein et al. reported about a production facility with a new scale-up approach for pure metal nanoparticles. The scale-up approach is the parallelization of multiple transferred arcs in one reactor, which were previously individually optimized. Furthermore, a novel filtration and bagging system is introduced, which is designed to handle pyrophoric metal nanoparticles. It is shown that the production rate of the process scales linearly with the number of transferred arcs, while the particle size stays on the nanoscale [215]. During scale-up of laboratory method, sometimes the desired features of nanoparticles are lost. For example, in a study of scale up of nanoparticle prepared using emulsion method, it was observed that increase in impeller speed and agitation time, particles size was decreased although entrapment efficiency was not altered [216]. Moreover, one of the important aspects for scale-up production of nanoparticles is the cost of the finished product and also consumable market [217].

### Nanoparticles and their toxicity

One of the limitations is the biocompatibility and potential toxicity issues of metal nanoparticles. Metal nanoparticles have been shown to have a cytotoxic effect on cells in vitro. This can be a concern when using metal nanoparticles for cancer treatment, as they may also kill healthy cells in addition to cancer cells. In addition, metal nanoparticles have been shown to have a genotoxic effect, which means they can damage DNA and potentially increase the risk of cancer [218]. These toxic effects need to be carefully considered and minimized in order to ensure the safety of metal nanoparticle-based treatments.

Gold nanoparticles have been widely used in biomedical fields due to their various advantageous and properties such as tunable sizes, facile synthesis, easy modification and strong optical properties [219, 220]. The outcome regarding gold nanoparticle toxicity is as yet conflicting. Some researcher reported that these particles are biocompatible and have unimportant toxicity ( [221–224]), while others emphasized that they cause ROS leading to oxidative stress and are also highly toxic because of their physicochemical properties ( [225–228]).

Even though the increased use of silver nanoparticles provides many advantages in biomedical applications, their toxicological effects have explored. Silver nanoparticles can enter the body in different ways and accumulate in different tissues and organs, and passing the blood–brain barrier and eventually reaching the brain [229].

Compared with other nanosized metals, silver nanoparticles show greater toxicity regarding the enhanced synthesis of ROS and leakage of the enzyme lactate dehydrogenase. The toxicity of silver nanoparticles was reported to be dependent on many factors, including physical and chemical properties of silver nanoparticle, environmental conditions.The smallest sized silver nanoparticle displayed a greater ability to induce hemolysis and membrane damage than particles of other sizes [219]. Another toxicological study on silver nanoparticles revealed polyvinylpyrrolidone-coated silver nanoparticles on bacteria, viruses, microalgae, fungi and human and animal cells (including cancer cell lines) via a variety of viability and toxicological assays. It was discovered that biological systems from different taxonomies were inhibited with a concentration of silver nanoparticles at a similar magnitude [230].

Because of zinc oxide nanoparticle’s wide application its toxicity to humans have been showed [220, 231]. De Berardis et al. evaluated the physical properties and the toxicological effects of zinc oxide nanoparticles. They showed a significant decrease in cell viability, remarkable morphological changes, apoptosis induction via ROS production and IL-8 release after treatment with zinc oxide nanoparticles (5 µg/cm^2^) for 24 h on human colon carcinoma cells. Besides, higher concentrations (10, 20 and 40 µg/cm^2^) induced about 98% cytotoxicity, with a cell survival lower than 5%, already after 24 h of treatment [232].

### Potential future directions and areas for further research

There are several potential areas of further research that could help to advance the use of metal nanoparticles for the diagnosis and treatment of gastrointestinal cancer. Some possible areas of focus include:

Developing methods to optimize the size, shape, and surface chemistry of metal nanoparticles for cancer diagnosis and treatment. As mentioned above, different sizes, shapes, and surface chemistries of MNPs can have different effects on cancer cells and may be more or less effective for different types of cancer. Developing methods to optimize these factors could improve the effectiveness of metal nanoparticle-based treatments.

Investigating the mechanisms by which MNPs interact with cancer cells and the factors that influence their effectiveness. Understanding the underlying mechanisms of action of metal nanoparticles could help to optimize their use for cancer diagnosis and treatment and identify potential targets for combination therapies.

Evaluating the long-term safety and effectiveness of metal nanoparticle-based treatments. While metal nanoparticles show promise as a potential technique for gastrointestinal cancer diagnosis and treatment, it is important to carefully evaluate their long-term safety and effectiveness in order to ensure that they are safe and effective for clinical use. To this end, clinical studies in humans are needed to confirm the effectiveness of these treatments and to assess their safety and potential side effects.

Developing new methods for the delivery of MNPs to GI cancer cells. Currently, there are several challenges in delivering metal nanoparticles to cancer cells, including the need to overcome the body’s immune system and the challenges of targeting specific types of cancer cells. Developing new methods for the delivery of metal nanoparticles could improve their effectiveness and broaden their potential use.

Exploring the use of MNPs in combination with other cancer therapies. Combination therapies that incorporate metal nanoparticles with other cancer treatments, such as chemotherapy or radiation therapy, could potentially improve the effectiveness of these treatments and reduce the toxicity of treatment.

## Discussion and conclusion

Gastrointestinal cancers are common solid malignant tumors, accounting for high mortality rates worldwide. MNPs have emerged as a promising tool for addressing this challenge, with a number of unique properties that make them well-suited for cancer diagnosis and treatment. They have been extensively studied in biological projects because of appreciable and flexible physico-chemical features.

In diagnosis, metal nanoparticles can be used to enhance the sensitivity and specificity of imaging techniques, such as computed tomography or magnetic resonance imaging. MNPs have been shown to have a strong contrast effect on computed tomography images, enabling the early detection of cancer. They can also be used to deliver diagnostic agents directly to GI cancer cells, improving the accuracy of the diagnosis and minimizing the need for invasive procedures.

Metal NP-based technologies provide more selectivity and sensitivity compared to the present instruments for early illness detection accessible of gastrointestinal cancer in the clinic, or they provide whole new capabilities that are not possible with conventional techniques. Although the selectivity and sensitivity of biomarker detection is improving, the laboratory diagnostic is not adaptable with clinical practice. The unique and tunable optical properties of metal NPs have revealed high potential in the expansion of biosensing and bioimaging techniques. Among all metallic NPs, gold and silver NPs show the most exciting physical properties for biosensing [233]. The great sensitivity and non-invasiveness of AuNP-based optical biosensors make them particularly suitable for use as POC devices. In fact, AgNPs have drawn a lot of attention as very sensitive materials for plasmonic biosensors. Although AgNPs are less stable and biocompatible than AuNPs, they nonetheless provide highly sensitive plasmonic biosensors for early diagnoses, particularly for pandemic illnesses like COVID-19. Furthermore, the use of Au with Ag NPs in core@shell structures offers a protective shell, and improves the plasmonic response of the resulting colloids. Some metal NPs such as CdS and Pd NPs with catalytic and photocatalytic activity are suitable candidates for early detection of contaminants, H_2_O_2_ and biomarkers [234, 235]. A high sensitivity in early detection could well be attained through using metal oxide NPs because of their huge surface area, quick electron transfer kinetics, biocompatibility, and catalytic activity [236]. This potential has prompted the application of metal oxide NPs in the development of several biosensors for pathogens, biological molecules, and biomarkers. The created nanocomposite and nanohybride-based biosensors can offer fresh approaches to difficult analytical evaluation and detection problems. Moreover, the exclusive properties of nanocomposites with improved sensitivity and stability make them suitable for early diagnosis methods and real-time sensing in complex matrices. MOFs have porous structures with high surface areas, therefore composites containing MOFs with functional materials have been applied in biosensor uses. Additionally, the MOFs have beneficial fluorescence quenching capability to fluorescence-labelled DNA probes [233].

In treatment, metal nanoparticles can be used to deliver therapeutic agents directly to cancer cells, improving the effectiveness of treatment and minimizing the side effects associated with traditional chemotherapy. These NPs improve the therapeutic efficacy of anticancer drugs because of their high drug loading, surface chemistry, electrostatic charge, and photothermal activity. Metal nanoparticles can also be used to generate local heating, which can be used to kill GI cancer cells through a process known as thermal ablation. In addition, MNPs have been shown to have a photodynamic effect, which can be exploited to deliver photodynamic therapy to cancer cells.

In current review, we discuss a number of methods used to produce MNPs, including bottom-up approaches (solid-state methods, liquid-phase synthesis methods, gas-phase methods, and biological methods) and top-down approaches (mechanical milling, laser ablation, and sputtering). We also discuss current research on using metal nanoparticles in treating gastrointestinal malignancies.

Moreover, metallic NPs can be functionalized with different targeting moieties e.g., liposomes, antibodies, folic acid, transferrin, and carbohydrates. Complementary investigations have established that surface functionalization of NPs for therapeutic use has greater advantages in cancers such as gastrointestinal cancer when compared to non-surface functionalized NPs. This is because functionalized NPs can be characterized by higher selectivity and drug targeted delivery efficiency [237]. In order to overcome certain limitations associated with unfunctionalized NPs such as high cost, lack of specificity, and increased toxicity, there are different functionalization approaches employed in nanotechnology. Antibody mediated targeting is alternatively one of the most common and efficient approach for active targeting [238]. When functionalized with targeting ligands/ antibodies, NPs can target specific cancer cell receptors thus, making it easier for therapeutic components to accumulate within targeted tumor cells. Conceptually, the conjugation process of ligands/ antibodies onto the surface encapsulated liposomal NPs involves the fabrication of active targeting moieties with highly concentrated linking molecules (e.g., peptide linker). In most cases, these targeting moieties (e.g., monoclonal antibodies, folic acid, transferrin, and carbohydrates) interact with extracellular receptor present on the surface of many cancer cells [239, 240].

In conclusion, the ability of metal nanoparticle to detect a wide range of molecular signals and biomarkers in real-time is just what drives breakthroughs in early detection, diagnostics, prognostics, and therapeutic strategy. Cancer nanotherapeutics have overcome several shortcomings of conventional therapies, including nonspecific biodistribution, low water solubility, and poor bioavailability. It provides high sensitivity, specificity, and multiplexed measurement capacity. The main limitation of nanoparticles is their toxicity, which can be controlled by the route of administration or by surface modification, by coating them with biodegradable polymers.

It is important to note that the specific mechanisms by which metal nanoparticles act may vary depending on the type of metal nanoparticle and the specific cancer type and stage. However, preclinical and clinical trials of using MNPs for GI cancer therapy are being researched and early results are promising, further research is needed to optimize the use of MNPs for the diagnosis and treatment of gastrointestinal cancer and to identify the most effective approaches for specific cancer types and stages and to translate clinical research into practice.

This review highlighted the effects of metal nanoparticles used for gastric cancer treatment including gold, zinc, silver, iron oxide, and Lanthanide nanoparticles, for colorectal cancer treatment including gold and silver nanoparticles, and just gold nanoparticles pancreatic cancer and hepatocellular carcinoma treatment. Result of this study showed that metal nanoparticles have anticancer effects on GI cancer cells which could provide future powerful therapeutic approaches.

## Data Availability

Not applicable.
